# Giant Retrorectal Epidermoid Cyst Masquerading as a Perianal Swelling

**DOI:** 10.1155/2020/5750382

**Published:** 2020-03-18

**Authors:** Reem Al-Shoura, Haifaa Malaekah, Waddah Al Bassam

**Affiliations:** ^1^Surgical Department, King Abdullah Bin Abdulaziz University Hospital, Princess Nourah Bint Abdulrahman University, Riyadh, Saudi Arabia; ^2^Radiology Department, King Abdullah Bin Abdulaziz University Hospital, Princess Nourah Bint Abdulrahman University, Riyadh, Saudi Arabia

## Abstract

A retrorectal epidermoid cyst is an uncommon congenital lesion that arises from the remnants of the embryonic tissues. This type of cyst is difficult to diagnose before surgery. In this study, we report a rare case of a giant retrorectal epidermoid cyst in a 30-year-old woman. Initially, the condition was diagnosed as a perianal abscess and treated with incision and drainage. Since the abscess recurred, a pelvic magnetic resonance imaging was ordered, which revealed an 8.2 cm perianal cyst with appearance not compatible with an abscess. Postsurgical histologic analysis confirmed a retrorectal epidermoid cyst. Postoperative course was uneventful, and the woman was discharged on postoperative day 3. She was doing well at 4 months of follow-up. This report suggests that retrorectal cysts should be considered in cases of recurrent perianal swellings/abscesses.

## 1. Introduction

Epidermoid cyst is one of the slow-growing benign subcutaneous lesions that may be observed throughout the body [1]; however, it is seldom found in the retrorectal space. Diagnosis of retrorectal epidermoid cyst is not established yet as the cysts are found incidentally in most of the cases, possibly due to the vague clinical presentation of the cyst [2]. The common presentation is related to the mass effect of the cyst. Most of the late presentations are because of the misdiagnosis with other presacral cysts such as tailgut, dermoid, and teratomas [3]. Complications associated with retrorectal cysts include perianal abscess, perineal fistula, rectal fistula, and bleeding in the rectum [4]. Here, we report a rare case of a retrorectal epidermoid cyst that presented as a perineal mass and was initially diagnosed as a perianal abscess.

## 2. Case Presentation

A 30-year-old woman, gravida 2 para 2 with a recurrent perianal abscess, was referred to the outpatient surgical department in our hospital for further evaluation and treatment. The woman had a history of perianal pain and swelling that were treated as a perianal abscess. She had undergone incision and drainage of the swelling 4 weeks before her visit to our hospital. She complained of increasing perineal swelling and discomfort over the last 6 months and denied symptoms of fever, constipation, perianal discharges, or other gastrointestinal symptoms. Her menstrual cycle was regular without abnormal uterine bleeding. The rest of the systemic review and her medical history and family history were unremarkable. Physical examination revealed normal vital signs; no signs of fever were observed. Abdominal examination was unremarkable. Pelvic examination revealed a large protruding mass at the natal cleft of the perineal area with no signs of inflammation that were appreciated like redness, warmth, or tenderness. The mass was of cystic consistency and the size was around 6 cm. The mass occupied the area between the anus and coccyx. The digital rectal exam revealed a round nontender soft mass in the posterior wall with a regular shape. Pervaginal examination was unremarkable. Laboratory investigation showed normal counts of total and deferential white cells and normal level of hemoglobin.

**Figure 1:**
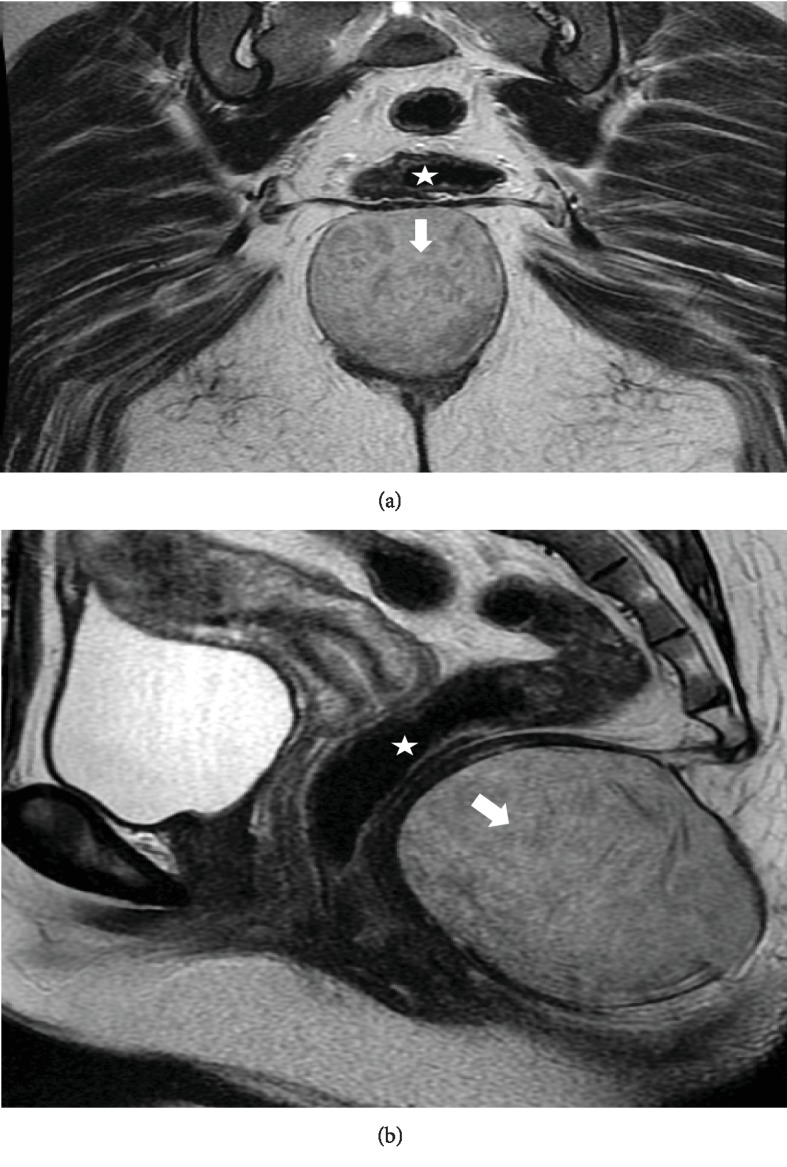
Perineal epidermoid cyst in a 30-year-old woman. Mass-oriented magnetic resonance imaging showing (a) coronal T2-weighted images (T2WI), and (b) sagittal T2WI. The lesion is indicated with a white arrow. T2WI demonstrated a well-circumscribed homogeneous structure located in the middle of the perianal region and posterior to the rectum (star); the lesion has an intermediate to high signal intensity, suggesting a fluid containing the cystic lesion. In unshown images, the lesion is hypointense relative to surrounding fat tissue on T1WI with no enhancement after gadolinium administration, only slight peripheral rim enhancement.

Magnetic resonance imaging (MRI) of the pelvis showed a 4.2 × 8.2 × 6 cm cystic lesion posterior to the anorectal canal and indenting the levator ani posteriorly. The lesion had a very thin rim of peripheral enhancement and minimal fat stranding posteriorly toward the natal cleft. It exhibited heterogeneity on a T2-weighted MRI image in homogenous fluid signals without restricted diffusion, solid component, or communication with the anorectal canal. Other pelvic organs were unremarkable (Figure 1). Based on the MRI results, the working diagnosis was epidermal inclusion cyst, tailgut cyst, or infected congenital cyst.

**Figure 2:**
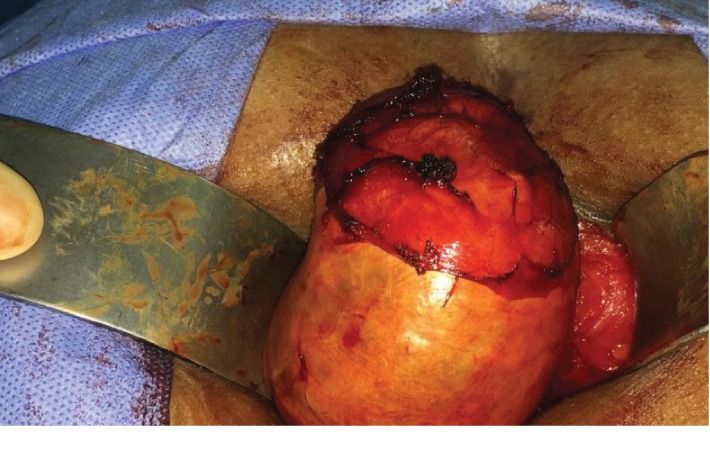
Initial rectal examination suggested a 10 × 4 × 6 cm retrorectal epidermoid cyst. The cyst was located between the anus and coccyx.

**Figure 3:**
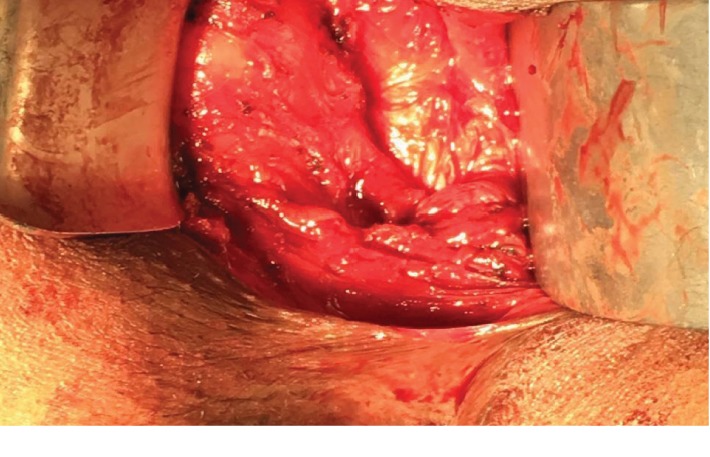
Excision of the cyst. A posterior transperineal approach was used to excise the cystic mass.

Hence, based on these clinical and imaging findings, a decision to operate was made. The surgical excision of the mass was performed posteriorly through a 5 cm midline perineal incision. The woman was placed in the prone jackknife position, revealing the swelling more prominently (Figure 2). During the procedure, a spine surgeon was consulted to avoid contact with the spinal cord. The mass was dissected from the wall of the rectum anteriorly and from the curve of the coccyx posteriorly (Figure 3). Complete resection of a 10 × 6 × 4 cm mass could be achieved without rupture. Histological analysis suggested an epidermoid cyst. Postoperative course was uneventful, and the patient was discharged on postoperative day 3.

## 3. Discussion

Majority of the retrorectal lesions originate as developmental masses. The lesions can be classified into congenital, inflammatory, neurogenic, osseous, and miscellaneous lesions [5]. Retrorectal epidermoid cysts are very rare to be seen, and most of the reported cases showed that these masses are found in females of reproductive age. However, there are 3 reported cases of presacral epidermoid cyst in adult males [2].

Epidermoid cysts are very slow-growing masses; consequently, many of them are asymptomatic [1]. The early symptoms that occur due to the mass effect of the cyst include constipation, pain, hydronephrosis, and difficulty in defecation [6]. Due to late clinical presentation of the cyst, complications may occur, which include infections and bleeding; however, the epidermoid cysts are unlikely to undergo malignant transformation [6]. Epidermoid cysts may be misdiagnosed with anorectal abscess, complicated fistula, and pilonidal sinuses [3]. In our case, the woman presented with perianal abscess, and surgical drainage was performed initially. Because of the rarity of the condition, the diagnosis of retrorectal epidermoid cyst could not be established clinically. Limited evidence is available on postoperative diagnosis of epidermoid cyst. According to medical literature, ultrasound or computed tomography may not be considered the best procedure for preoperative diagnosis of epidermoid cysts, as these procedures may result in nonspecific findings [2]. MRI is considered more accurate for the diagnosis of an epidermoid cyst. Recent reports recommend using MRI with diffusion-weighted images as preoperative diagnostic modality for retrorectal/presacral cyst [2, 6]. Classically, epidermoid cysts show a hypointense mass on T1-weighted images and hyperintense mass on T2-weighted images, indicating diffusion restriction. Because of the presence of keratin, some hypointensity foci may be seen on T2-weighted images [2]. Uncomplicated cysts may show a regular flat contour without enhancement [7]. MRI may also help differentiate the epidermoid cyst from other retrorectal/presacral masses including teratomas, tailgut, or infected congenital cyst, which usually show a homogenous pattern on MR imaging [6]. In this case, MRI was used as the first imaging modality. Definitive diagnosis of epidermoid cyst could be achieved by the histopathological examination. Typical histological findings of epidermoid cysts include a wall with a thin line of squamous epithelium, and the cyst is filled with keratinized material [6]. Our observation in the current case is consistent with the previous findings. The definitive treatment of retrorectal epidermoid cyst is complete surgical excision [8]. Many surgical approaches can be applied such as anterior, posterior, combined, or laparoscopic approaches [8]. The choice of approach is based on the size, location of the mass, and available facilities at the treatment center [9]. Anterior abdominal approach is applied when the tumor is above the midbody of S3, while posterior approach (transsacral, parasacral, or transperineal) is used when the mass is located under the level of the midbody S3, without the involvement of any adjacent tissue or organ [8]. Combined method is used only for suspected invasion to adjacent structures, or if the mass is slightly above S3 [8]. In our case, the cyst was excised by transperineal approach.

The feasibility of the laparoscopic approach is controversial, especially in solid tumors [9]. A previous study showed the results of the laparoscopic approach for retrorectal tumors in 12 patients with no major postoperative complications [9]. The only relative contraindication of the laparoscopic approach over open approach in a retrorectal cyst is the risk of malignancy and spillage in case of perforation [4]. To avoid recurrence and complication, complete excision of the cyst wall is needed.

A retrorectal epidermoid cyst is a rare entity, which should be considered when encountering a patient with recurrent perianal swellings/abscesses. The current case report contributes to the current medical literature by providing evidence on the importance of MRI in the diagnosis of an epidermoid cyst. We highly recommend using MRI as an initial diagnostic modality for early detection and differentiation of the epidermoid cyst from other pelvic lesions. Findings of the current study may significantly help clinicians achieve better treatment outcomes in treating retrorectal epidermoid cyst.
